# Supplementary material for: Long-Term Sex- and Genotype-Specific Effects of 56Fe Irradiation on Wild-Type and APPswe/PS1dE9 Transgenic Mice
Source: Int J Mol Sci. 2021 Dec 10;22(24):13305. doi: 10.3390/ijms222413305 (PMC8703695; doi:10.3390/ijms222413305)
Supplement: Supplementary file 1 [file ijms-22-13305-s001.zip › ijms-1502147-supplementary.pdf]

Supplementary Figure S1: ^{56}Fe irradiation did not affect general health or body weight of mice.

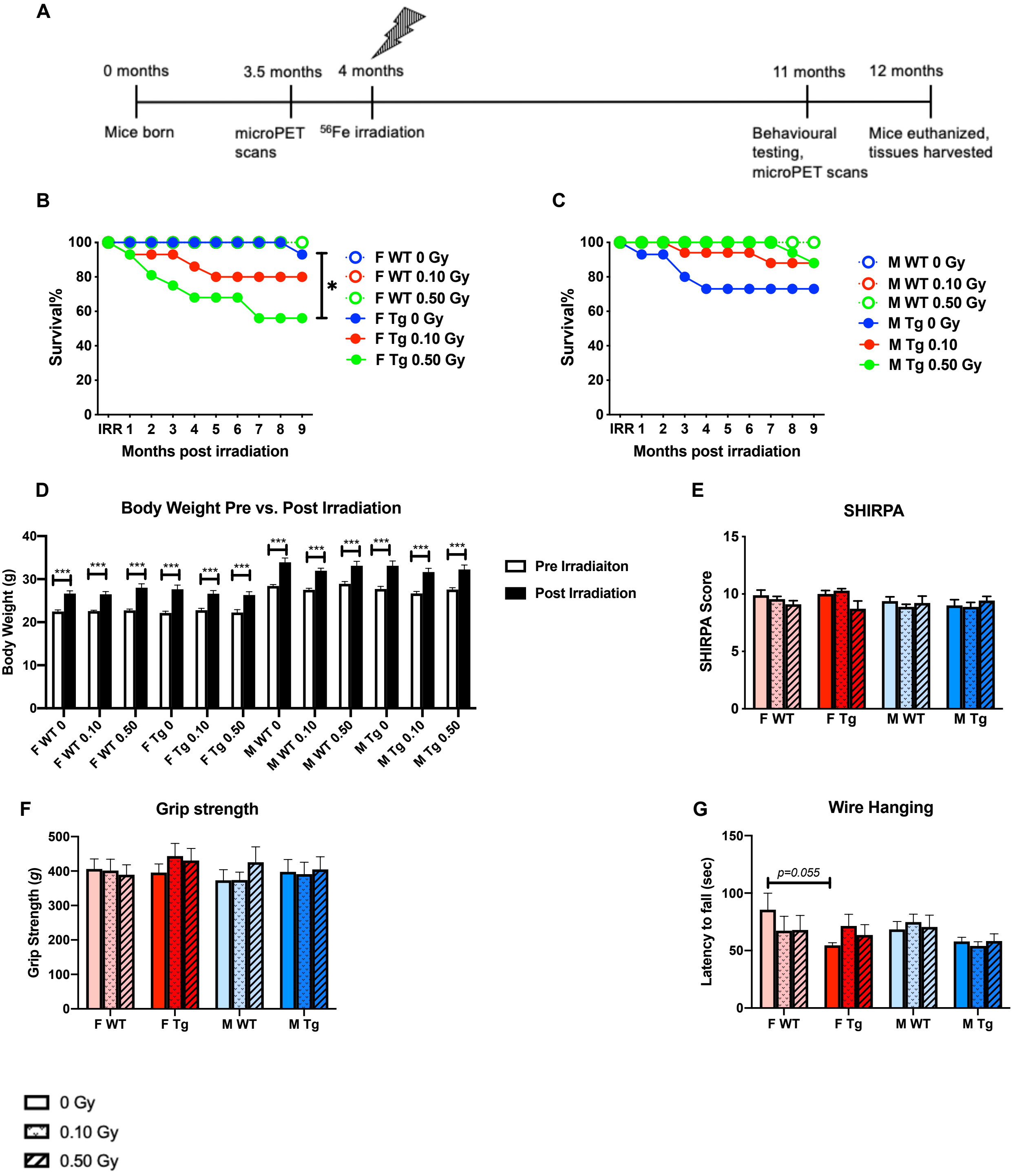

Supplementary Figure S2: ^{56}Fe irradiation affected mostly female WT mice on startle response but increased pre-pulse inhibition in APP/PS1 males.

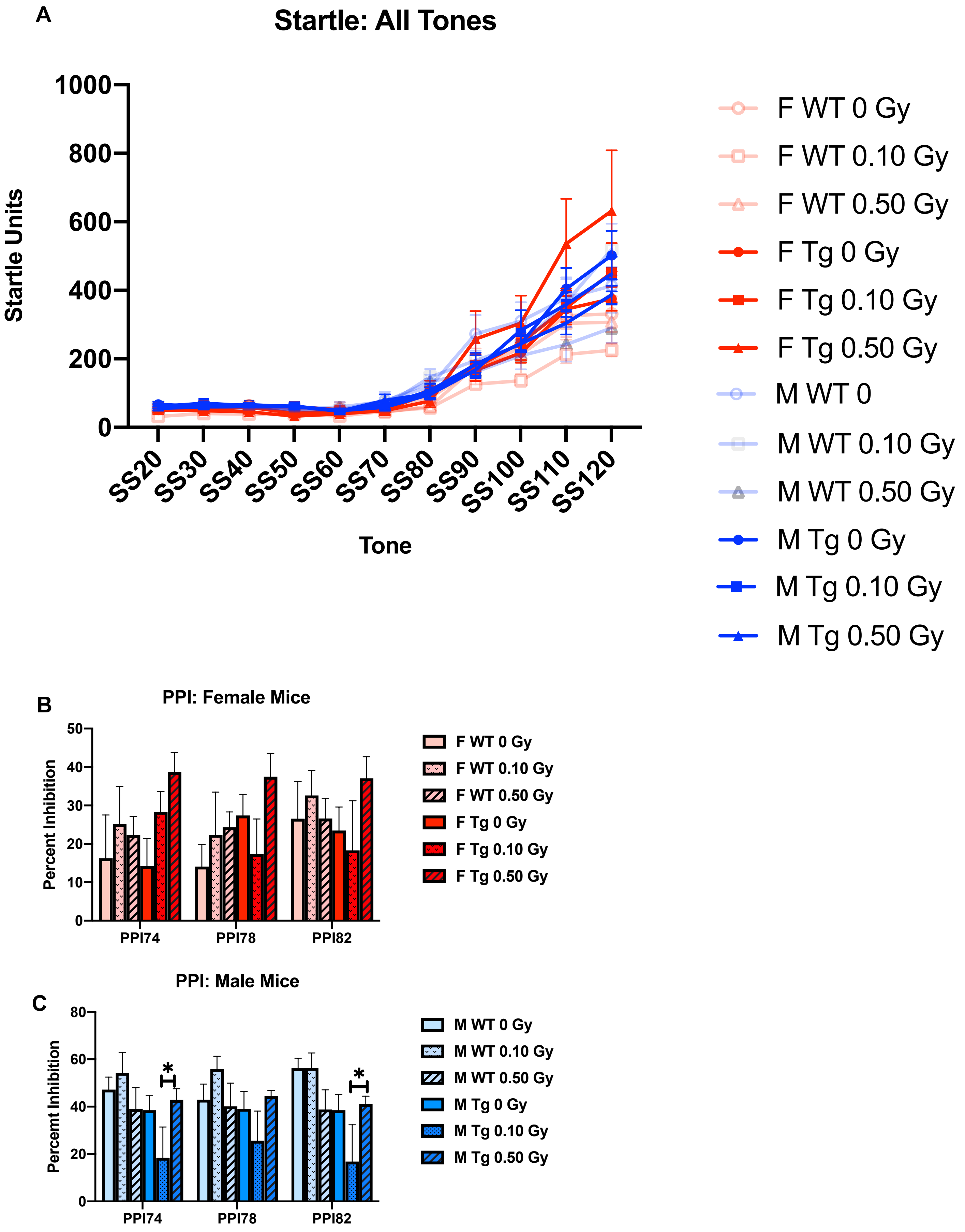

Supplementary Figure S3: ^{56}Fe irradiation did not affect whole brain expression of synaptic markers in mice.

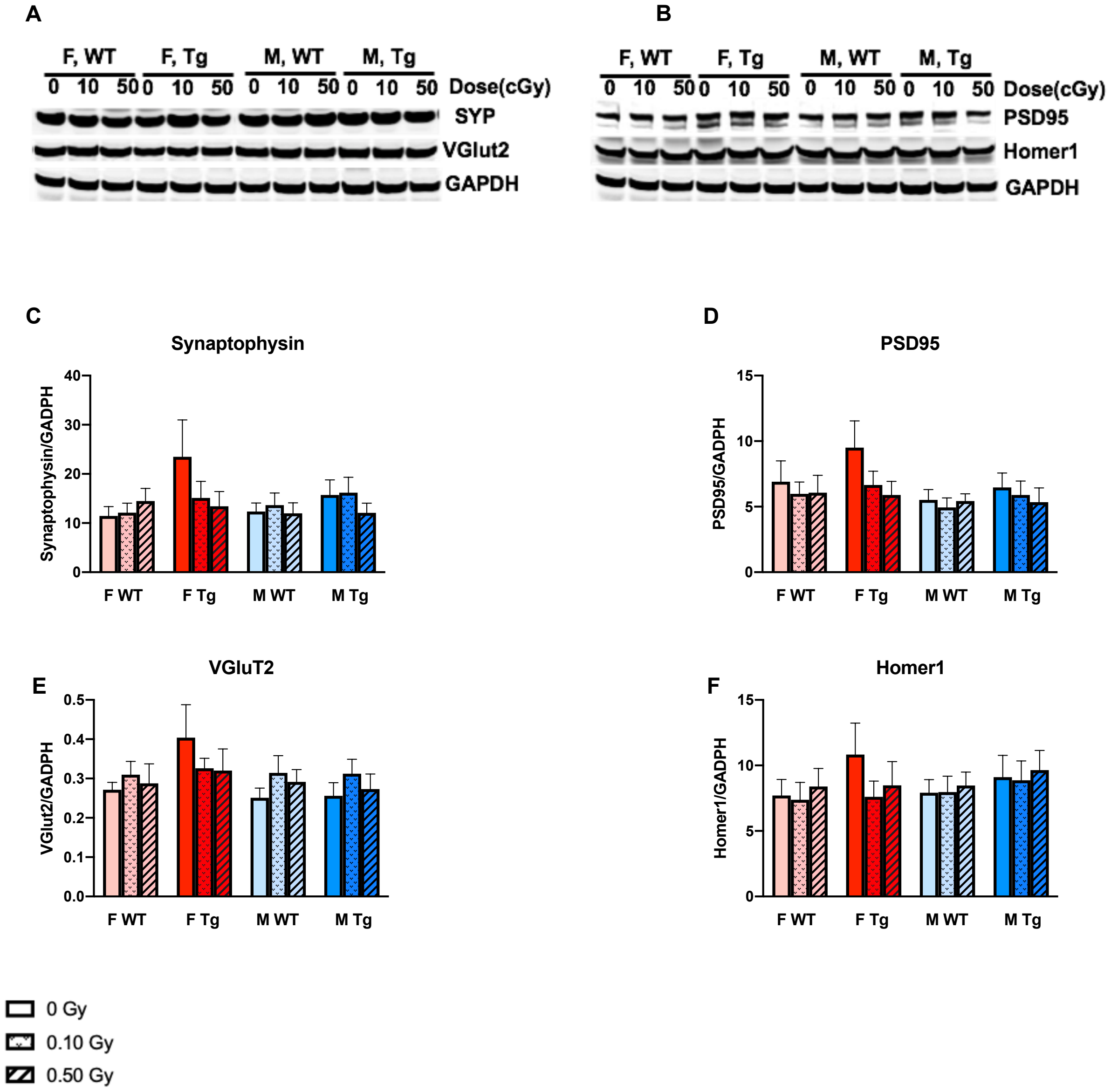

Supplementary Figure S4: ^{56}Fe irradiation did not affect hippocampal and cortical expression of synaptic markers.

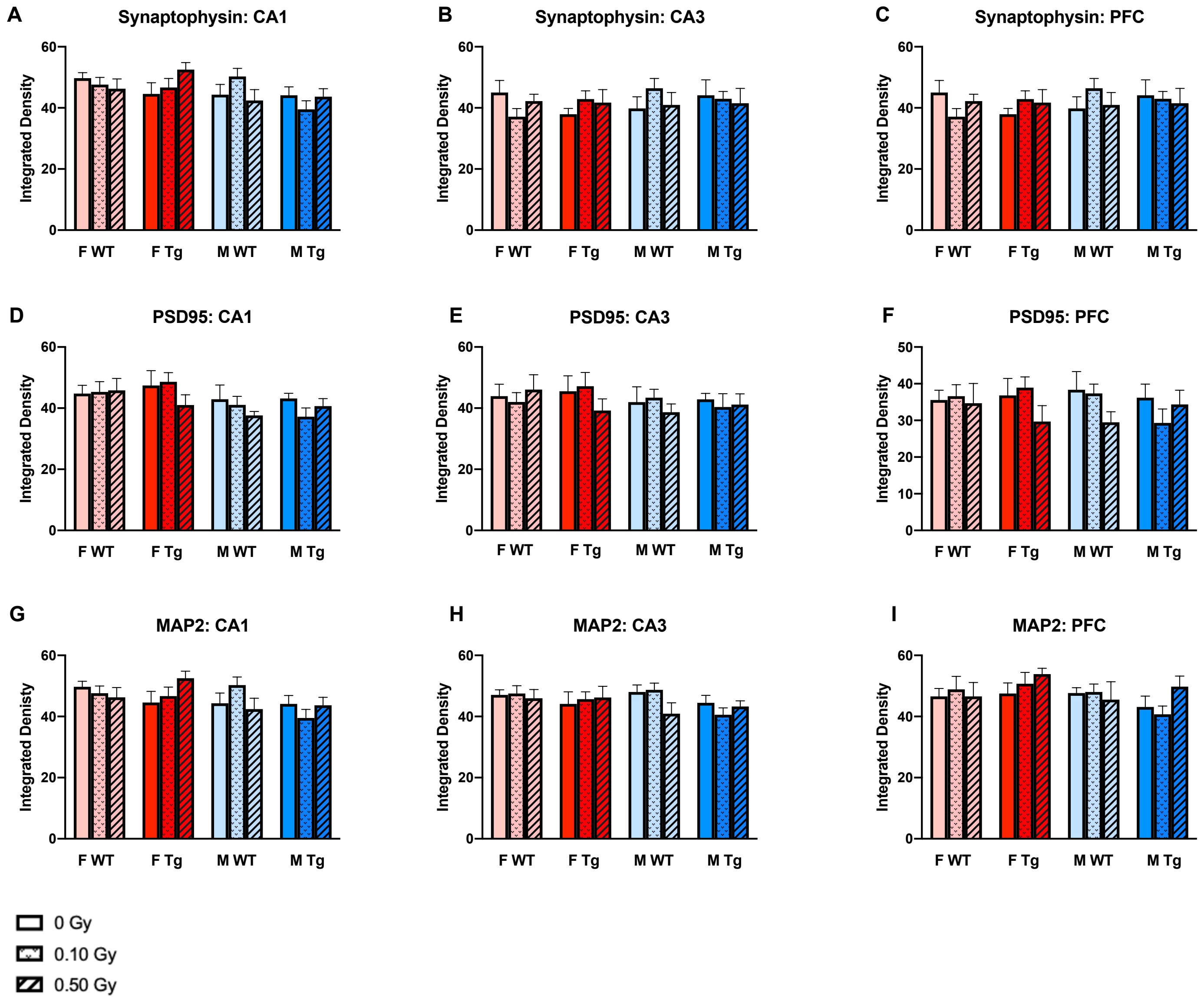

Supplementary Figure S1. ^{56}Fe irradiation had late effects on mouse survival rate but did not affect mouse body weight or strength. A schematic of the experimental timeline is shown in (**a).** Female APP/PS1 mice receiving 0.50 Gy of radiation had significantly lower survival rates than sham-irradiated female APP/PS1 mice (**b**). Survival rates did not differ between any of the male mice (**c**). Mouse body weight was measured prior to shipment and irradiation (pre-irradiation), and again at 8 months after irradiation (post-irradiation). All sex and genotype groups were significantly heavier 8 months after irradiation, but irradiation did not body weights of any group (**d**). There were no differences between groups on the SHIRPA test (**e**). There were little to no effects of ^{56}Fe irradiation on grip strength (**f**), though sham-irradiated female WT mice trended towards increased endurance on the wire hanging test compared to sham-irradiated female APP/PS1 mice (**g**). *n*=12-18 mice/group. Survival curve data was analyzed with a Log-rank (Mantel Cox) test. Body weight data was analyzed with a 2-way repeated measures ANOVA. SHIRPA data was analyzed with a 3-way ANOVA.

Supplementary Figure S2. ^{56}Fe irradiation had little to no late effects on sensory reactivity or sensory gating. ^{56}Fe irradiation altered startle reactivity mostly in females, (**a,** significance bars not shown). At 110 dB, female APP/PS1 mice in the 0.50 Gy group startled significantly more than female APP/PS1s in the sham and 0.10 Gy groups. At 120 dB, female APP/PS1 mice in the 0.50 Gy group startled significantly more than sham-irradiated APP/PS1 females while WT males in the 0.10 Gy group startled significantly more than those in the 0.50 Gy group. At 90 dB, sham-irradiated WT males startled significantly more than WT males in the 0.50 Gy group. ^{56}Fe irradiation only altered PPI in males (**b-c**). Data were analyzed with 3-way ANOVAs followed by 1-way ANOVAs of male and female mice with Fisher's Protected Least Significant Difference (PLSD) post hoc test.

Supplementary Figure S3. No late effects of ^{56}Fe irradiation on brain levels of synaptic markers. **a-b,** Representative western blot images for pre-synaptic markers, synaptophysin and VGluT2 (**a**) and post-synaptic markers, PSD-95 and Homer1 (**b**). **c-f:** whole brain expressions of Synaptophysin (**c**), PSD-95 (**d**), VGluT2 (**e**), and Homer1 (**f**), were measured by Western blot using mouse brain homogenates (*n*=7-9 mice/group). No late irradiation effects on synaptic markers were found. *: $p<0.05$. Data were analyzed by 3-way ANOVAs followed up with 1-way ANOVAs with Tukey comparisons and planned, unpaired, 2-tailed *t* tests between sham groups as necessary. Non-parametric data was analyzed with the Kruskal Wallis and/or Mann Whitney U tests

Supplementary Figure S4. No late effects of ^{56}Fe irradiation on brain region-specific expressions of synaptic markers, Synaptophysin and PSD-95, or dendritic marker, MAP2. Immunofluorescent labeling of Synaptophysin, PSD-95 and MAP2 were performed on fixed mouse brain cryosections. The immunofluorescent integrated density of each marker was analyzed in hippocampal CA1 and CA3 regions, as well as in prefrontal cortex (PFC). **a-c:** region-specific analysis of Synaptophysin in hippocampal CA1 (**a**), CA3 (**b**) and prefrontal cortex (**c**). **d-f:** region-specific analysis of PSD-95 in hippocampal CA1 (**d**), CA3 (**e**) and prefrontal cortex (**f**). **g-i**: region-specific analysis of MAP2 in hippocampal CA1 (**g**), CA3 (**h**) and prefrontal cortex (**i**). We found no late irradiation effects on either marker in any brain regions. *n*=7-9 mice/group, 2 sections/mouse/marker. Data were analyzed by 3-way ANOVAs followed up with 1-way ANOVAs with Tukey comparisons and planned, unpaired, 2-tailed *t* tests between sham groups as necessary. Non-parametric data was analyzed with the Kruskal Wallis and/or Mann Whitney U tests.
